# In-vivo corneal confocal microscopy: Imaging analysis, biological insights and future directions

**DOI:** 10.1038/s42003-023-05005-8

**Published:** 2023-06-19

**Authors:** Jeremy Chung Bo Chiang, Maitreyee Roy, Juno Kim, Maria Markoulli, Arun V. Krishnan

**Affiliations:** 1 School of Optometry and Vision Science, Faculty of Medicine and Health, University of New South Wales, Sydney, NSW Australia; 2 School of Optometry, College of Health and Life Sciences, Aston University, Birmingham, NSW UK; 3 School of Clinical Medicine, University of New South Wales, Sydney, NSW Australia; 4 Department of Neurology, Prince of Wales Hospital, Sydney, NSW Australia

**Keywords:** Peripheral nervous system, Microscopy

## Abstract

In-vivo corneal confocal microscopy is a powerful imaging technique which provides clinicians and researcher with the capabilities to observe microstructures at the ocular surfaces in significant detail. In this Mini Review, the optics and image analysis methods with the use of corneal confocal microscopy are discussed. While novel insights of neuroanatomy and biology of the eyes, particularly the ocular surface, have been provided by corneal confocal microscopy, some debatable elements observed using this technique remain and these are explored in this Mini Review. Potential improvements in imaging methodology and instrumentation are also suggested.

## Abstract (web summary)

This Mini Review provides a focus on corneal confocal microscopy and insights into the neuroanatomy of the corneal surface using this technique and open debates are highlighted.

## Introduction

The ocular surface is richly innervated with sensory nerves, particularly at the cornea which is a clear dome-like tissue forming the frontmost part of the eye. In-vivo corneal confocal microscopy is a technique which is capable of imaging corneal microstructures at different depths of the tissue. Together with immunohistochemical techniques, it has provided insight into biological functions of different neuronal and immunological constituents of the cornea. The sub-basal nerve plexus has garnered substantial interest as it is the densest and most homogenous of the several nerve plexi in the cornea, situated between the basal epithelial layer and Bowman’s layer^1^. Corneal confocal microscopy has provided an opportunity to image corneal microstructures non-invasively and monitor how these change overtime with exposure to various stimuli. This Mini Review summarises current iterations of corneal confocal microscopy instrumentation with particular focus on laser scanning confocal microscopy which is the most widely used form. In addition to the optics of confocal microscopy and image analysis, this Mini Review also explores the anatomical, neurobiological and immunological insights afforded by corneal confocal microscopy as well as the pervasive gaps in our knowledge. Limitations and future directions in the advancement of the corneal confocal microscopy technique will also be explored (Box 1).

Box 1 Current applications and potential future improvements of the corneal confocal microsocpy technique

**Table:** 

Most established current applications	∙ Clinical settings: Aiding in the diagnosis of ocular surface diseases, particularly in identifying the causative pathogen in infectious keratitis such as fungal^63^ or acanthamoeba keratitis^90^
	∙ Clinical research settings: Potential use as a clinical diagnostic tool in identifying patients with peripheral neuropathy due to a range of aetiologies, particularly diabetes^70,71^
Potential improvements	∙ Artificial intelligence (AI) in the analysis of features identified in corneal confocal microscopy images, especially the sub-basal nerve plexus
	∙ Enhancing the user friendliness of the instrument or technique (e.g. precise localisation or eye tracking capabilities, wider field of imaging, non-contact procedures)

### Emergence and optics of confocal microscopy

Ocular imaging techniques have advanced and improved over the past few decades, with in-vivo confocal microscopy emerging as a potential diagnostic technique for ocular surface diseases due to its ability to observe ocular surface microstructures in a non-invasive and rapid manner. This imaging technique is based on the principle of confocal microscopy first described in 1955^2^. Compared to conventional light microscopy, confocal microscopy produces images with higher resolution and better out-of-focus information rejection. It is also able to capture images of cellular layers from various depths within a thick tissue specimen using its optical sectioning capability and hence, is well suited for the investigation of intact tissue in living organisms.

**Fig. 1:**
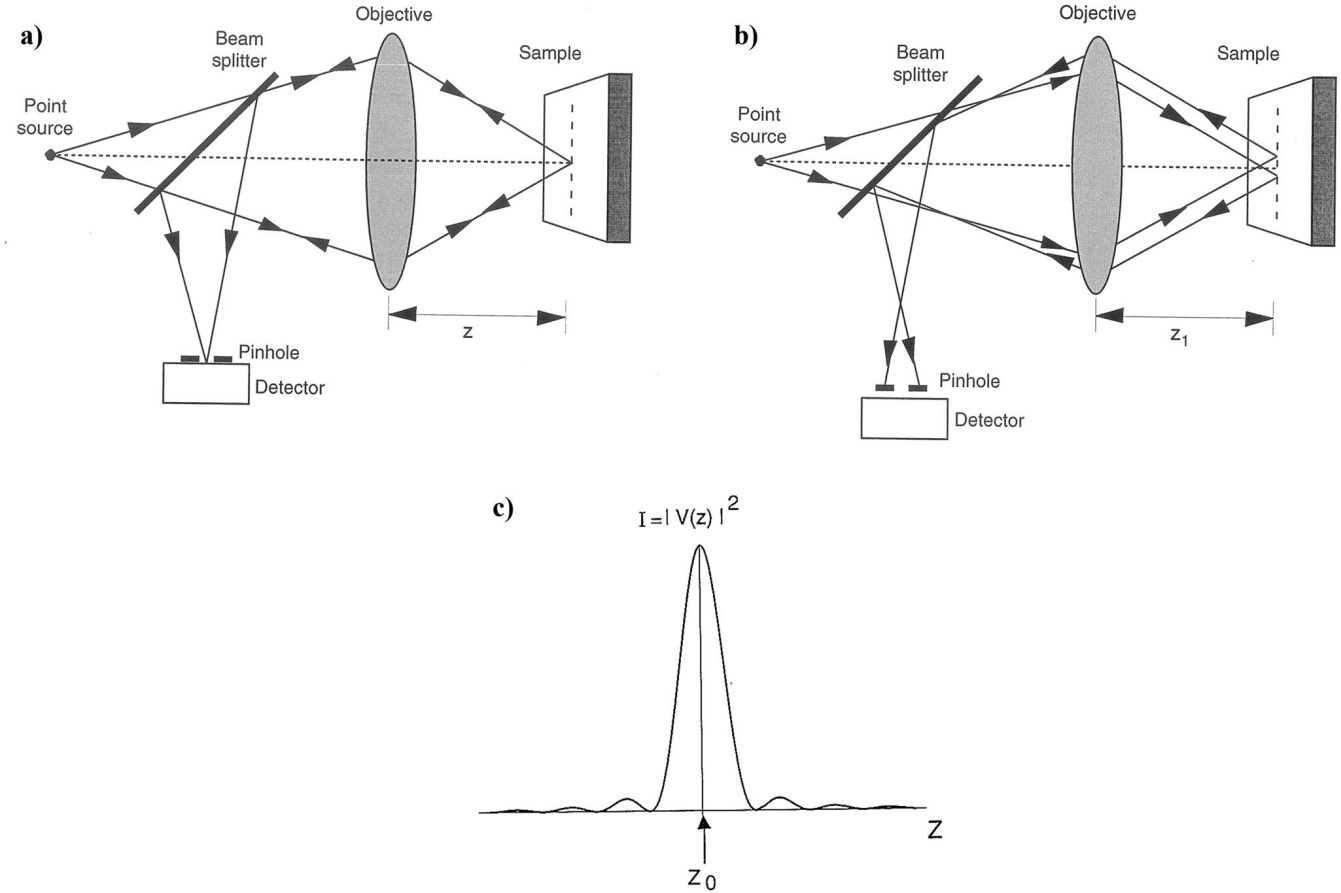
Principle of confocal microscopy. Schematic diagram of a confocal microscope illustrates the optical section property (**a**) when the sample is placed at the correct image distance (z), the reflected light is focussed on to the point detector, and (**b**) when the sample lies at some other image distance (z_1_), the reflected light focuses above or below the plane of the point of detection. **c** The V(z) profile represents the axial resolution of a confocal system. A narrower axial response width indicates a higher axial resolution.

The optical sectioning property of the confocal microscope makes it possible to store a three-dimensional data set of intensity values of thick objects by using a point source and a point detector in the illumination and detection paths, respectively^3^. The sample is illuminated using a diffraction-limited focused laser spot and the reflected or transmitted light is detected by a point-like detector (Fig. 1). When light comes from the focal region of a specimen, it is focussed onto a point detector (Fig. 1a) and hence produces a strong signal. However, light which comes from a region away from the focal plane (say z_1_) is defocused at the pinhole (Fig. 1b) and therefore produces a much weaker signal. In terms of the resolution of the image, a narrower axial response width implies a higher axial resolution. In other words, the narrower the peak in the V(z) profile, the better the system is at distinguishing between objects located at different axial positions (Fig. 1c). This unique system contributes to the powerful imaging capabilities of the in-vivo corneal confocal microscope in producing images of high resolution and magnification.

### Confocal imaging of the ocular surface

There are three types of commercially available in-vivo confocal microscopy developed for assessing the ocular surface: the Tandem Scanning Confocal Microscope (Tandem Scanning Corp, Virginia, USA), the Confoscan 4 (Nidek Technologies Srl, Padova, Italy), and the Heidelberg Retinal Tomograph with Rostock Corneal Module (HRT-RCM, Heidelberg Engineering, GmBH, Dossenheim, Germany). These microscopes differ from one another in terms of the intensity of the light source, magnification, image contrast, and image resolution. However, the HRT-RCM which uses a laser scanning confocal microscope provides higher resolution and magnification compared to earlier corneal confocal microscopy models^4^, and is currently more widely used by clinicians and researchers worldwide.

While confocal microscopy is capable of imaging various regions of the ocular surface, it has been more widely used to characterise features of the cornea. Corneal epithelial cells, stromal keratocytes and endothelial cells can be imaged at high resolution which enable assessment of their integrity and shape. Cell densities, including resident immune cells, can also be monitored using corneal confocal microscopy^5,6^.

Nerve fibres innervating the cornea form several plexi within anterior layers of the cornea, from the stroma to the epithelium. As the sub-basal nerve plexus is the densest and most homogenous of these nerve plexi, it has been widely studied particularly in ocular surface diseases. Currently, quantifying parameters such as corneal nerve fibre length or density are normally conducted using software which could include manual tracing or methods such as CCMetrics^7^, and semi-automated tracing such as NeuronJ, a plugin in ImageJ (National Institutes of Health, Maryland, USA). Automated procedures have also been proposed to substantially reduce the time needed to analyse corneal nerve parameters, although studies have shown that conventional automated procedures may underestimate these parameters compared to manual or semi-automated procedures^8,9^. The following section outlines further advancements in the analysis of corneal nerve parameters particularly with artificial intelligence (AI).

### Image analysis

Advances in AI have encouraged clinical neuroscience towards more objective systems for quantifying corneal nerve morphology from corneal confocal microscopy images^10–12^. Automation provides clinicians with diagnostic leverage for inferring disease from measurable image structure using computational models developed in software. Two of the most popular geometric attributes that have been modelled using AI previously include nerve density or length and tortuosity of the sub-basal nerve plexus.

Nerve density is commonly defined as the standardised distribution of nerve fibres over a finite area of image space in mm/mm^2^ or the ‘sum of the nerve branches observed within a frame’^13^. Models that automatically estimate nerve density require images of corneal nerve fibres to be fully segmented. Any model fragmentation generated by false negatives would lead to underestimates of nerve density.

Scarpa et al.^12^ used simple convolutional Gabor kernel filters in conjunction with image equalisation to enhance the nerve contours in corneal confocal microscopy images to improve nerve segmentation. They found the estimates of segmented nerve length from their model were correlated with the lengths of subjectively identified nerve contours, accounting for around 74% to 88% of subjective judgments of nerve length. Another recent approach used a multiscale dual-model approach that applied even-symmetric Gabor filters to the image in one step to then enhance contours and a background noise reduction operation in an alternate step^14^. Even symmetric Gabor filters model the behaviour of simple cells found in the primary visual cortex and thus model subjective gradings of corneal nerves. The success of this approach has been improved with the support of artificial neural networks^15^ and has been leveraged upon by end-user software packages like ACCMetrics^16^.

Kim and Markoulli^17^ devised a ‘structure enhancement’ model based on multiscale spatial Gabor filters and operations to eliminate vignetting of corneal confocal microscopy images. Although this approach is simple and principled in modelling human visual detection of image contours, it does not outperform ACCMetrics^17^. However, another recent proposal used U-Net convolutional neural networks to perform corneal nerve segmentation from corneal confocal microscopy images^18^. The sensitivity of their model performed at the level expected of human subjective graders, which has proved useful for segmenting corneal nerve contours in corneal confocal microscopy images to estimate nerve density. While deep learning algorithms continue to evolve and be developed to enhance nerve segmentation and evaluation, most are limited to research settings and require further validation before implementation in clinical practice^19,20^. A software using a customised deep learning-based approach known as deepNerve has recently been developed and used for animal and clinical human studies^21,22^, demonstrating the promising potential of artificial intelligence in this area.

In comparison to nerve density, nerve tortuosity is not as well defined. Oliveira‐Soto and Efron^13^ proposed a grading system whereby a score of zero indicates nerve contours appear straight and a score of four indicates nerve contours frequently and largely change orientation across their length. A potential limitation of this definition is there is no clear guide on what an extreme example of tortuosity should look like and how intermediate levels perceptually scale. However, the advent of machine learning provides the ability to ‘inversely’ determine the image-based parameters that predict human observer gradings.

Using a generative model design, ref. ^10^ proposed a reliable automated model of tortuosity that was motivated by the need to account for the overall magnitude of changes in nerve orientation across the image and number of local ‘twists’—denoted by the number of inflection points along a segmented nerve fibre’s length. An alternative approach used Multinomial logistic ordinal regression to show that mean curvature at two different spatial scales was diagnostic of human subjective tortuosity gradings^23^. However, a more recent study by ref. ^24^ proposed that nerve tortuosity was best quantified directly using U-Net segmentation to assess adjacent angular detection of nerve contour orientations (USAAD). This model first obtains a local-scale index of tortuosity by determining the straight-line contours that connect every adjacent pair of nerve pixels segmented using the earlier-described U-Net segmentation algorithm. Reducing the number of key points allows the model to assess tortuosity over longer spatial ranges. This model was found to generate outstanding performance in accounting for subjective ratings. Evidence also suggested that subjective ratings of tortuosity is computed across multiple scales spanning the full range of nerve lengths available. Could this mean that tortuosity estimates are weighted towards longer nerves when they are visible? Consistent with this view, a simple model that weighted tortuosity estimates based on nerve fibre length was found to exhibit sound correspondence with human judgements of tortuosity and provided diagnostic leverage in the prediction of diabetes^11^.

In summary, there appears to be great benefit achieved thus far through image analysis techniques developed in previous literature. One potential limitation of these approaches is that ground truth is consistently referenced to subjective gradings made by human observers. However, this may not be a limitation as the end goal of AI is to optimally assist clinicians with their diagnostic and monitoring responsibilities, but not replace them.

### Neurobiological and immunological insights

Neural and immunological features are the two main components investigated using corneal confocal microscopy. As mentioned, the sub-basal nerve plexus is the one of the most studied part of the cornea given its uniform and dense nerve fibre distribution^1^. Both corneal confocal microscopy and immunohistochemical techniques have demonstrated distinct morphological patterns in this plexus, with some of the earlier montaged corneal confocal microscopy images showing nerve fibres converging towards a clockwise or anti-clockwise spiral^25^. This region has been termed the inferior whorl which is situated about 1–2 mm inferonasal from the anatomical centre of the cornea. While various theories have been suggested for the spiral pattern of the sub-basal nerve plexus, recent studies have shown that interactions with corneal epithelial cells also migrating from limbal regions have substantial impact on dictating the course of nerve fibre migration^26,27^. Specifically, axon guidance ligands have been found on corneal epithelial cells including Semaphorins, Ephrins and Netrins^28^. Various studies have also indicated the potential for the inferior whorl to act as a landmark for monitoring neuronal changes particularly in peripheral neuropathic conditions^29,30^. However, there has been evidence of deviations from this spiral pattern associated with advancing age^31^ or neuronal damage^32^ which may confound identification of the inferior whorl region. While laser scanning confocal microscopy can provide substantial detail of corneal microstructures in high resolution and magnification, one of its limitations is the small field of view associated with each image frame which constitutes an area of 0.16 mm^2^. In relation to quantitative sub-basal nerve plexus outcomes including corneal nerve parameters and dendritic cell density, various studies have shown that random sampling of a selected number of images (usually 8 or more) from a total pool collected from areas of interest of the cornea may be sufficient in optimising reproducibility and accuracy of measures^32–34^. Widefield imaging capabilities could further facilitate analysis of nerve migration^35,36^ and monitor neuronal morphological changes in the sub-basal nerve plexus.

Certain resident immune cells can also be imaged within the cornea through corneal confocal microscopy. This has been supported by earlier findings which identified the presence of bone marrow-derived CD11c^+^ dendritic cells which are potent antigen presenting cells located mainly within the epithelial and anterior stromal layer of the cornea, as well as macrophages in the stroma^37^. Plasmacytoid dendritic cells, a subpopulation of bone marrow-derived dendritic cells, have also been shown to be present particularly in the peripheral regions with pivotal roles in inducing immune tolerance^38^ or wound healing^39^. While corneal confocal microscopy lacks the capability of providing functional characterisation of immune cells, researchers have used its high resolution and magnification to analyse morphological properties particularly with dendritic cells. Generally, dendritic cells with larger size and/or more dendrites are characterised as activated or mature^40,41^, indicating a more inflamed state in the cornea.

The biological interconnectedness between both eyes can also be demonstrated through the cornea. Recent studies using corneal confocal microscopy to investigate corneal infections including herpetic keratitis or cataract surgery in one eye have shown bilateral reduction in corneal nerves in both eyes^42–44^. Experimentally-induced corneal nerve cut or injury to one eye in animal studies have also affected corneal nerve function^45^ and increased CD11c^+^ or CD11b^+^ dendritic cells expressing co-stimulatory molecules in the unaffected eye^46^. Such interdependence between the two ocular surfaces may partly be due to a neurogenic inflammatory reflex mediated by activation of the transient receptor potential vanilloid 1 channel involved in nociceptive signalling and subsequent substance P release^47^. A corneal-trigeminal axis involving upregulation proinflammatory cytokines, substance P and infiltration of immune cells may also contribute to the propagation of inflammation from the affected corneal surface to bilateral trigeminal ganglia^48^.

### Prevailing uncertainties in the identification of corneal microstructures

While corneal confocal microscopy has provided substantial information of anatomical structures and biological function in the cornea, certain gaps in knowledge remains pervasive. corneal confocal microscopy has been used to monitor corneal nerve recovery following therapeutic or surgical interventions in various ocular surface diseases^49,50^, but it is also thought to be able to detect signs of aberrant regeneration. Microneuromas are another neuronal feature that has been identified with corneal confocal microscopy and commonly described as terminal enlargements of a corneal nerve^51,52^. It is thought that these enlargements are associated with aberrant nerve regeneration and neuropathic pain following injury to the corneal nerve^53^. While severed nerves have historically demonstrated these abnormal neuronal growths^54^, recent studies have shown that some features previously identified as microneuromas with corneal confocal microscopy could potentially be corneal nerve stromal-epithelial nerve penetration sites^55^. The latter could be characterised as being diffuse hyperreflective sites as supported by immunohistochemical findings showing continuation between the sub-basal corneal nerve and underlying originating stromal nerve^55^. Emerging evidence from murine models demonstrated that the presence of these penetration sites may be elevated with metabolic stress or dysfunction^56^. However, standardisation in the identification of these neuronal features is still required.

Hyperreflective round cells have also been identified amongst the sub-basal nerve plexus particularly at the inferior whorl region. As their size and hyperreflectivity seem to reflect those of dendritic cells, they are considered to be a subtype of immune cells which lack dendrites. These cells have been given different labels including globular cells^57^, dot-like features^31^ or round-shaped immune cells^58^. The evidence for the true nature and biological significance of these cells remain limited with little knowledge of immunohistochemical correlates. However, CD86^+^ round-shaped dendritic cells closely associated with sub-basal nerve fibre branching points have been observed by ref. ^59^. These cells penetrate the basement membrane into the stroma and may play a role in guiding nerve movement or trajectory^59^. Whether these cells represent those observed in corneal confocal microscopy remain unknown.

Cells with dendritiform morphology are often labelled as dendritic cells in studies using corneal confocal microscopy. A recent study identified the presence of resident CD8^+^ memory T-cells in constant motility following a resolution of local infection which also demonstrated dendritic morphology using immunostaining techniques^60^. Highly motile dendritiform cells were also observed with corneal confocal microscopy in humans by constructing single images of the same area imaged by corneal confocal microscopy in a time series^60^. These are reminiscent of ‘immature’ dendritic cells labelled in previous studies^61,62^, however whether corneal confocal microscopy can distinguish these immune cell types based on morphology alone requires further investigation. It is evident that more widefield in-vivo corneal imaging techniques which can also localise corneal nerve regions to be imaged could facilitate more thorough comparisons and precise monitoring of these corneal microfeatures.

### Current clinical and research applications

**Fig. 2:**
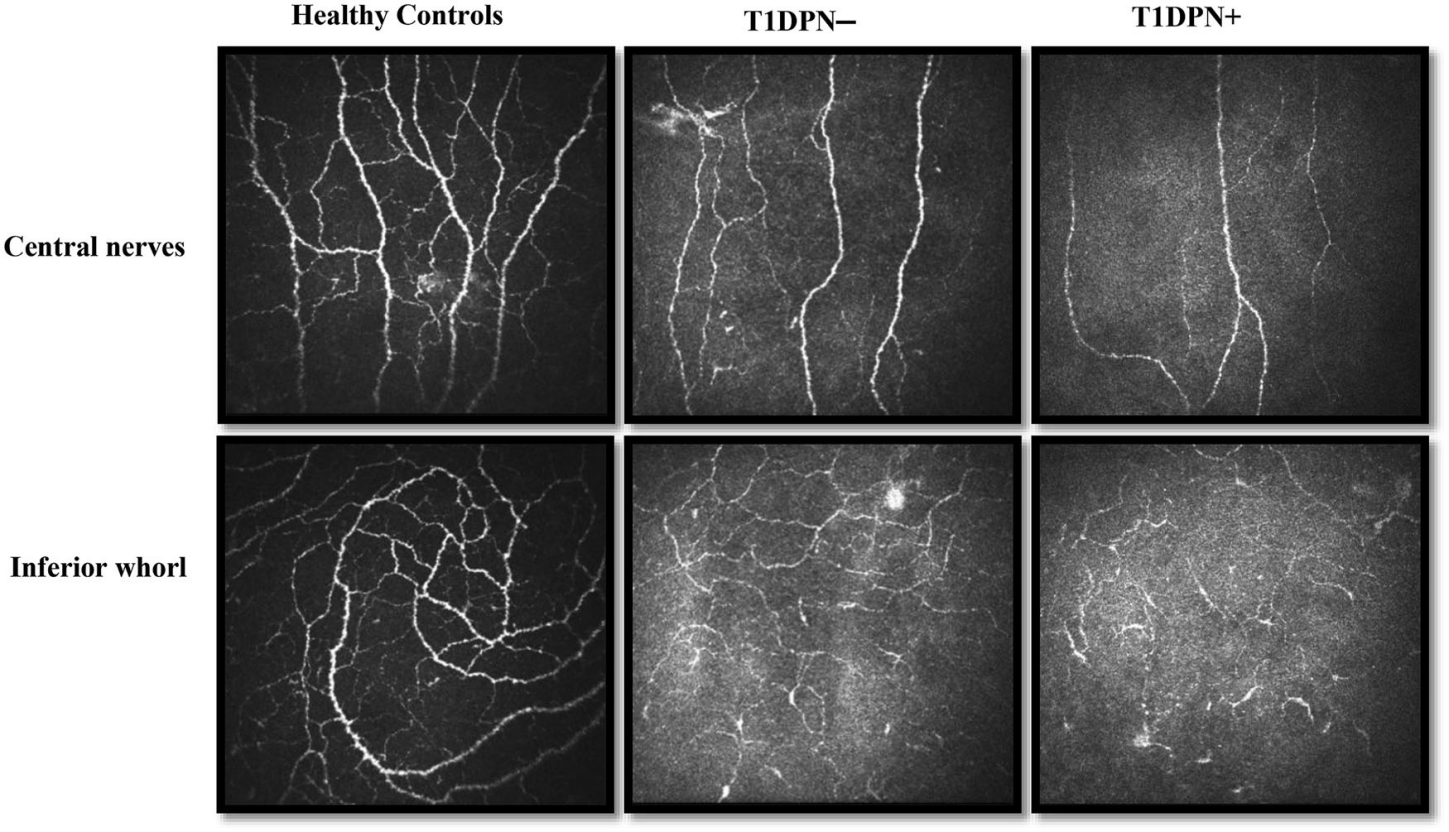
Representative images of the sub-basal nerve plexus at the central cornea and inferior whorl region with healthy controls, patients with type 1 diabetes without peripheral neuropathy (T1DPN–) and with peripheral neuropathy (T1DPN+). Note that the nerve density is the least in the T1DPN+ images, followed by the T1DPN– and then healthy controls. The figure was reprinted from Tummanapalli et al. with permission from Elsevier^72^.

In specialty clinics for corneal disease, corneal confocal microscopy has demonstrated high discriminative ability in distinguishing infectious keratitis of various aetiologies. It is particularly useful in cases with atypical clinical presentations where differentiation is more difficult such as the visualisation of fungal filaments and acanthamoeba trophozoites or cysts to confirm an uncertain diagnosis^63–66^. Recent findings in corneal confocal microscopy have also been associated with systemic biological health such as higher corneal nerve parameters with higher serum levels of omega-3 polyunsaturated fatty acids particularly docosahexanoic acid^67^ and associations between reduced corneal nerve parameters in Alzheimer’s disease or transgenic mice overexpressing human non-mutated tau^68^. Corneal confocal microscopy has mostly been studied in the context of diabetes, which is one of the most common causes of peripheral nerve injury in the distal extremities particularly the feet^69^. Its utility in diagnosing and predicting the development of diabetic peripheral neuropathy has been demonstrated in previous studies^70–72^ (Fig. 2). Loss of corneal nerves and increase in corneal dendritic cells were found to be more prominent in affected patients^73,74^. Similar changes have been observed in ocular surface conditions particularly in dry eye disease^75^ which is known to have elements of neurological and inflammatory dysfunction^76^.

### Future outlook

Several improvements could be made to enhance imaging capabilities of the corneal confocal microscopy, a few of which have been discussed in previous sections of this Mini Review. The most commonly used methodology in clinical research settings involves random selection of a set number of images with sufficient quality from a pool of images obtained from a participant’s eye prior to analysis^33,34^. Sampling strategy of adequate images has been shown to produce sufficient accuracy, however for longitudinal or clinical monitoring purposes, real-time widefield imaging capabilities may provide more insight into regional changes in the cornea over time or following exposure to various stimuli^77,78^. In fact, a minimum repeatable area of 1.5 mm^2^ is shown to have reliable morphological characterisation of the sub-basal nerve plexus^79,80^.

Precise and repeatable localisation technology built into corneal confocal microscopy instrumentation, akin to those available in optical coherence tomography (OCT) to facilitate follow-up assessments of the posterior pole, is yet to be devised for routine clinical use. More recently, OCT technology with real-time eye tracking has been adapted to provide non-contact visualisation of corneal nerves^81^, as well as other ocular surface structures in a larger field of view including tear film patterns, limbal crypts and conjunctival vasculature^82,83^. However, these techniques are yet to be adopted for widespread use and the inherent curvature and clarity of the cornea continue to present a challenge in widefield imaging. Volumetric scans may also shed more light on 3-dimensional structures particularly in terms of the physical interactions between neuronal, epithelial and immune elements. This may further inform the concept of peripheral epineuroimmune interactome which characterises the biological interdependence of these microstructures essential for the maintenance of health^84^. In order to enhance patient- and clinician-friendliness, non-contact procedures would also be desirable to prevent deterrence of patients who may be more sensitive to mechanical sensations^85^. AI is also increasingly utilised to further improve the sophistication of corneal nerve^20,86–88^ or immune cell segmentation and detection^20,89^. These advancements may improve the applicability of corneal confocal microscopy and also improve efficiency in observing corneal microstructures to achieve the aim of understanding their biological significance.

### Reporting summary

Further information on research design is available in the Nature Portfolio Reporting Summary linked to this article.

## Supplementary information

Reporting Summary
